# Pharmacokinetics and anti‐nausea effects of intravenous ondansetron in hospitalized dogs exhibiting clinical signs of nausea

**DOI:** 10.1111/jvp.13087

**Published:** 2022-07-28

**Authors:** Cindy K. Sotelo, Sarah B. Shropshire, Jessica Quimby, Sydney Simpson, Daniel L. Gustafson, Kristin M. Zersen

**Affiliations:** ^1^ Department of Clinical Sciences, College of Veterinary Medicine and Biomedical Sciences Colorado State University Fort Collins Colorado USA; ^2^ Department of Veterinary Clinical Sciences, College of Veterinary Medicine and Biomedical Sciences The Ohio State University Columbus Ohio USA

**Keywords:** half‐life, nausea, ondansetron, pharmacokinetics

## Abstract

The purpose of this study was to evaluate the pharmacokinetics of intravenous (IV) ondansetron in a population of hospitalized dogs exhibiting clinical signs of nausea. The causes of nausea included pancreatitis, gastroenteritis, endocarditis, chemotherapy‐induced nausea, diabetes mellitus and ketoacidosis, acute kidney injury with aspiration pneumonia, pyometra, uroabdomen, neoplasia, and hepatopathy. Twenty‐four dogs were randomly assigned to one of the following IV ondansetron protocols: 1 mg/kg q12h, 0.5 mg/kg q12h, 1 mg/kg q8h, 0.5 mg/kg q8h. Serum was collected at 0, 0.25, 0.5, 1, 2, 4, 8, 16, and 24 h after the first dose, and nausea scores were recorded at multiple time points. Ondansetron and arginine vasopressin (AVP) concentrations were measured via high‐performance liquid chromatography coupled to tandem mass spectrometry and ELISA, respectively. Noncompartmental pharmacokinetic modeling and dose interval modeling were performed. Ondansetron displayed linear pharmacokinetics. In the 0.5 mg/kg group, mean *C*_max_ = 214 ng/ml, AUC_0–8h_ = 463 ng/ml*h, and calculated half‐life was 1.9 h. In the 1 mg/kg group, mean *C*_max_ = 541 ng/ml, AUC_0–8h_ = 1057 ng/ml*h and calculated half‐life was 1.6 h. Serum ondansetron concentrations were not significantly different between dogs that required rescue anti‐nausea medication (non‐responders) and dogs that did not require rescue therapy (responders). In total, 83.3% of patients in the 0.5 mg/kg q8h, 0.5 mg/kg q12h, and 1 mg/kg q8h groups had improvement in nausea scores. In total, 66.7% of patients in the 1 mg/kg q12h group had improvement in nausea scores. In total, 33% of patients had resolution of nausea in the 0.5 mg/kg q8h, 1 mg/kg q8h, and 1 mg/kg q12h groups, and 16% of patients had resolution of nausea in the 0.5 mg/kg q12h group. AVP concentrations were highly variable and did not correlate with nausea scores. Nausea scores significantly decreased regardless of dosage protocol. AVP was not a reliable biomarker of nausea in this group of dogs.

## 1 INTRODUCTION

Ondansetron is a 5‐HT_3_ receptor antagonist given intravenously to treat nausea and vomiting in veterinary patients. Intravenous (IV) administration of ondansetron has been shown to be an effective anti‐emetic in dogs with parvoviral infection, dogs with vestibular disease, dogs given single dose emetogens, and dogs undergoing single dose chemotherapy protocols (Foth et al., 2021; Kenward et al., 2017; Sedlacek et al., 2008; Seynaeve et al., 1992; Sullivan et al., 2018).

Empirical dosing for IV ondansetron in dogs ranges from 0.5 to 1 mg/kg every 8 to 12 h (Plumb, 2019), but this is not based on pharmacokinetic data in dogs exhibiting naturally occurring clinical signs of nausea and has only been reported for patients undergoing single dose chemotherapy or toxin injection (Baek et al., 2015; Kenward et al., 2017; Kretzing et al., 2011).

Diagnosing nausea in clinical patients is based on clinical signs and physical examination findings; however, this assessment is subjective. Clinical signs of nausea may include lip‐licking, hypersalivation, refusing food, and vomiting. Multiple studies have attempted to reduce the subjective nature of assessing nausea by implementing nausea scores (Foth et al., 2021; Kenward et al., 2015; Sedlacek et al., 2008; Sullivan et al., 2018). Other studies aimed to assess nausea by measuring arginine vasopressin (AVP) concentrations, which has been shown to increase after dogs were given the chemotherapeutic agent cisplatin, a known emetogen (Cubeddu et al., 1990; Kenward et al., 2017). Additionally, ondansetron limited the increase in AVP concentrations following cisplatin administration and prevented emesis (Kenward et al., 2017).

The purpose of this study was to evaluate the pharmacokinetics and anti‐nausea effects of IV ondansetron in a population of hospitalized dogs with clinical signs of nausea.

## 2 MATERIALS AND METHODS

### 2.1 Sample size calculation

The sample size calculation was made using PK as the primary outcome with a serum ondansetron variability of 60%, which was based on an oral ondansetron pharmacokinetic study in cats (Quimby et al., 2014), and to show a difference of twofold based on a dose increase of twofold. For *p* = .05 and a power of .8, we required six animals per dosing group (total of 24 patients divided into four different dosing protocols).

### 2.2 Animals

This prospective randomized study was approved by the Colorado State University Clinical Review Board, and all owners gave informed written consent to participate. Patients were enrolled over a period of 6 months. To be eligible for the study, dogs must have been at least 1 year of age, be at least 5 kg, and have a nausea score of at least 2, using a previously validated nausea scoring system (Table 1; Foth et al., 2021; Kenward et al., 2015, 2017). Dogs were excluded if a small intestinal mechanical obstruction was documented or suspected or if any anti‐nausea medications had been administered within 48 h of enrollment. All dogs underwent a physical examination and had a complete blood count, chemistry panel, and appropriate imaging modalities (abdominal radiographs and/or abdominal ultrasound) performed to assess for underlying causes of the clinical signs of nausea. Dogs were defined as non‐responders if rescue anti‐nausea medications were administered to alleviate clinical signs of nausea during the study period. Dogs were defined as responders if they did not require rescue therapy.

**TABLE 1:** Nausea scoring system in dogs

Behavior	Description	Score
Salivation	Drooling, increase in swallowing frequency, gulping, chewing movements	1
Gastrointestinal disturbances	Pica, belching, productive or nonproductive vomiting	1
Lip smacking	Lip licking, licking the nose, nictitation (winking)	1
Excitement behaviors	Apprehension, restlessness, rapid breathing	1
Vocalization	Murmuring, groaning, whining	1
Withdrawal behaviors	Standing with head drooping, closing eyes, yawning, drowsiness, lethargy, and excessive sleeping	1
Appetite	Decreased appetite, avoidance of food bowl	1

*Note*: Table references from Kenward et al. (2015, 2017).

### 2.3 Ondansetron dosing

Peripheral IV catheters were placed in all patients at the time of hospitalization. Dogs were randomized into one of four ondansetron dosing groups: 0.5 mg/kg IV q12h, 0.5 mg/kg IV q8h, 1 mg/kg IV q12h, or 1 mg/kg IV q8h (2 mg/ml, injectable). There were six dogs enrolled in each group.

All other medications were prescribed at the discretion of the primary veterinarian; however, additional anti‐nausea medications were not prescribed at the time of hospitalization. If a patient continued to demonstrate signs of nausea after administration of ondansetron, the primary veterinarian could prescribe rescue anti‐nausea medications.

### 2.4 Blood sampling

Blood samples were collected at the following timepoints: 0, 0.25, 0.5, 1, 2, 4, 8, 16, and 24 h after administration of the first dose of ondansetron. Serum was collected by centrifugation after clot formation and frozen in aliquots at −80°C for batched analysis to assess ondansetron and AVP concentrations.

### 2.5 Scoring clinical signs of nausea

Nausea scores were assigned by a veterinary technician or veterinarian using a previously validated nausea score (Table 1). The evaluators were not blinded to the patient dosing/frequency group. In patients dosed every 8 h, nausea scores were assigned at baseline, 4 h, and 8 h after the administration of the first dose. In patients dosed every 12 h, nausea scores were assigned at baseline, 4 h, and 12 h after the administration of the first dose.

### 2.6 Materials

Ondansetron was collected from West/Ward Pharmaceuticals (Eatontown, NJ, USA) and Accord Healthcare (Durham, NC, USA) purchased through the Colorado State University Veterinary Teaching Hospital. An ELISA kit for the analysis of canine arginine vasopressin was purchased from MyBioSource (MBS1604344, San Diego, CA).

### 2.7 Sample collection

Blood was collected in standard red top tubes without additives. All samples were centrifuged (5000× *g* for 5 min), and the serum was separated and stored in a polypropylene microcentrifuge tube at −80°C until processing for ondansetron or AVP analysis.

### 2.8 Ondansetron analysis in serum

Ondansetron analysis in canine serum samples was performed using a liquid chromatography coupled to tandem mass spectrometry (LCMS) assay based on a previously published method (Quimby et al., 2014). Standard and quality control (QC) samples were prepared by adding 5 μl of known amounts of ondansetron dissolved in methanol:MilliQ water (1:1) and 5 μl of the internal standard (IS) zolpidem (50 ng/ml in methanol:MilliQ water (1:1)) to 50 μl of blank canine serum followed by mixing. Unknown samples were prepared by adding 5 μl of methanol:MilliQ water (1:1) and 5 μl of the IS mixture followed by mixing. Standard, QC, and unknown samples were then extracted by the addition of 500 μl pentane: ethyl acetate (1:1) with 0.1% ammonium hydroxide followed by mixing on a vortex shaker for 10 min. Samples were then centrifuged for 10 min at 13,300× *g* followed by 400 μl of the organic phase (top) being transferred to a fresh microcentrifuge tube and evaporated to dryness on a rotary evaporator operated at medium heat. The samples were then reconstituted in 100 μl methanol:MilliQ water (1:1) with 0.1% acetic acid and transferred to auto‐sample vials with glass low volume inserts. Samples were then analyzed using an ABI 3200 QTrap triple quadrupole mass spectrometer with an Agilent 1200 LC system and HTC‐Leap autosampler. Sample volume injected was 10 μl and chromatography performed using a Waters Phenyl 2.5 μmm 4.6 × 50 mm LC column with a Phenomenex C18 filter frit guard cartridge. The mobile phase consisted of a gradient system using methanol and 10 mM ammonium acetate with 0.1% acetic acid with starting concentrations of 10% methanol that was increased linearly from 1.0 to 2.0 min to 98% methanol where it was held until 3.5 min when the initial concentration of 10% methanol was reestablished linearly over 0.5 min and held for the remainder of the 5.0 min run. Ondansetron and the IS (zolpidem) were detected by multiple reaction monitoring under positive ion mode of ondansetron at 294.3 *m/z* → 170.2 *m/z* and zolpidem at 308.3 *m/z* → 235.2 *m/z* using unit resolution for Q1 and Q3, ion spray voltage of 5500 V, source temperature of 500°C, curtain gas of 35, ion source gases 1 and 2 at 60, and collision gas at medium. Compound‐dependent parameters (DP, depolarizing potential; EX, exit potential; CEP, collision entrance potential; CE, collision energy; and CXP, collision exit potential) were optimized using instrument optimization algorithms. The lower limit of quantitation was 0.9 ng/ml. The accuracy and precision were 95.6 ± 3.4 at the low QC (3.9 ng/ml), 93.1 ± 4.7 at the mid ranges (15.6 and 62.5 ng/ml), and 93.2 ± 5.7 at the high end with 24/28 (86%) QC samples showing an accuracy >85%.

### 2.9 Arginine vasopressin (AVP) analysis in serum

Serum AVP concentrations were measured using an ELISA assay kit from MyBioSource (MBS1604344; Pirrone et al., 2019). The manufacturer's protocol was followed for sample and analysis with absorbance read at 450 nm on a Bio‐Tek plate reader.

### 2.10 Dose interval modeling

Prediction of ondansetron in canine serum following dosing of IV ondansetron was performed using nonparametric superposition based on serum samples from the first 24 h. The pharmacologically active serum concentration of ondansetron that prevents emesis in canine patients was extrapolated from data in a previous study that states that placebo patients exhibited nausea behaviors between hours 3.5 and 4.75 h after an emetogen was given and the patients given ondansetron IV had lower nausea scores between this time with ondansetron concentrations of 10 ng/ml (Kenward et al., 2017). In this population, IV ondansetron was given to dogs as a single dose of 0.5 mg/kg following a single dose of cisplatin. In the placebo group, nausea scores increased 3 h after cisplatin injection. In the dogs that received ondansetron, plasma ondansetron concentrations remained above 10 ng/ml, which reduced nausea scores and prevented increases in AVP concentrations. (Kenward et al., 2017). Simulation of multiple dosage regimens was done using concentration versus time data for 24 dogs dosed at 0.5 and 1.0 mg/kg and Nonparametric Superposition using Phoenix 64 v8.3.4.295 (Certara, Inc).

### 2.11 Pharmacokinetic and statistical analysis

The ondansetron serum concentration versus time data for each dog was subject to non‐compartmental analysis using Phoenix WinNonLin v 8.3.4.295 (Certara, Princeton NJ). AUC values were calculated using the linear trapezoid method with linear interpolation. Values were considered significantly different if the *p*‐value was <.05.

Normality of data was assessed with a Shapiro–Wilk test. Differences between responders and non‐responders were compared using a Fisher's exact test. Nausea scores at 4 h and 8 or 12 h depending on dosage protocol for all dogs were compared with baseline using Friedman test with Dunn's multiple comparison test. Differences between groups for duration of time above 10 ng/ml were compared using Tukey's multiple comparisons test. All statistical analysis was performed using Prism Software (Prism 9; GraphPad, La Jolla, CA, USA).

## 3 RESULTS

### 3.1 Animals

Twenty‐four dogs hospitalized at the Colorado State University Veterinary Teaching Hospital were included in the study. The patient population included 13 males (eight castrated, five intact) and 11 females (10 spayed and one intact). Breeds varied with five Labrador retrievers or Labrador mixes, three golden retrievers, three American Staffordshire terriers mixes, and one each of the following: Boykin spaniel, Brittany spaniel, bichon frise, Shih Tzu, Maltese, Chihuahua, French bull dog, Irish Wolfhound, English Setter, Giant Schnauzer, Australian Shepherd, Rottweiler, and a mixed breed dog.

Underlying diseases varied in our population of patients. Seven patients were presented for vomiting and diarrhea with presumed gastroenteritis, three patients had diabetic ketoacidosis, two had pancreatitis, two had suspected hepatopathies (one possible bacterial cholangiohepatitis and one was liver failure of suspected congenital origin), one of the following etiologies was suspected/diagnosed: uroabdomen, pyometra, gastric mass, aspiration pneumonia, endocarditis, and post chemotherapy reaction. Four patients had multiple etiologies contributing to their illness including one patient with pancreatitis and a urinary tract infection, one with pancreatitis and aspiration pneumonia, one with regurgitation of unknown origin and aspiration pneumonia, and one with pancreatitis, aspiration pneumonia, and acute kidney injury. The volemic and hydration status of the patients varied.

### 3.2 Ondansetron concentrations

All dogs achieved measurable serum concentrations of ondansetron. Noncompartmental pharmacokinetic modeling variables are presented in Table 2. Ondansetron displayed linear pharmacokinetics.

**TABLE 2:** Pharmacokinetic parameters in hospitalized dogs exhibiting signs of nausea receiving intravenous ondansetron at either 1 or 0.5 mg/kg

PK parameter	0.5 mg/kg dose	1.0 mg/kg dose
AUC_0–8h_ (ng/ml*h)	463 ± 392 (53–1370)	1057 ± 962 (188–3448)
CL (L/h/kg)	1.98 ± 2.37 (0.30–9.30)	2.07 ± 1.60 (0.24–5.27)
*C*_max_ (ng/ml)	214 ± 130 (32–563)	541 ± 477 (168–1660)
*V*_z_ (L/kg)	4.36 ± 4.39 (1.28–17.78)	4.32 ± 3.82 (1.12–11.65)
MRT (h)	1.7 ± 0.6 (1.1–3.2)	1.4 ± 0.6 (0.6–2.6)
*t*_1/2_λ (h)	1.9 ± 1.0 (1.1–4.9)	1.6 ± 0.7 (1.0–3.3)

*Note*: Values represent the mean ± standard deviation with the range shown below in parentheses. Pharmacokinetic parameters were estimated by non‐compartmental analysis using Phoenix WinNonlin software (Certara, Inc.).

### 3.3 Dose interval modeling

**FIGURE 1:**
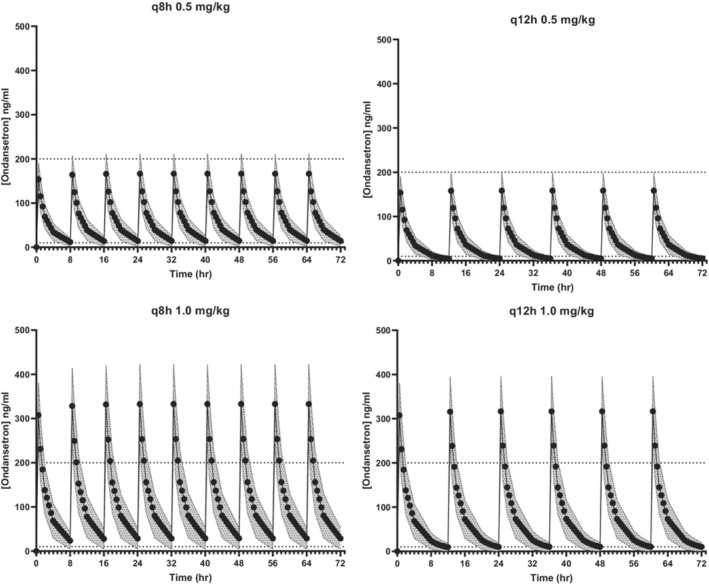
Dose interval modeling of 0.5 and 1 mg/kg ondansetron administered in hospitalized dogs exhibiting signs of nausea intravenously every 8 and 12 h (*n* = 24). The area between the dotted lines represents the estimated effective serum concentration of 10–250 ng/ml.

**FIGURE 2:**
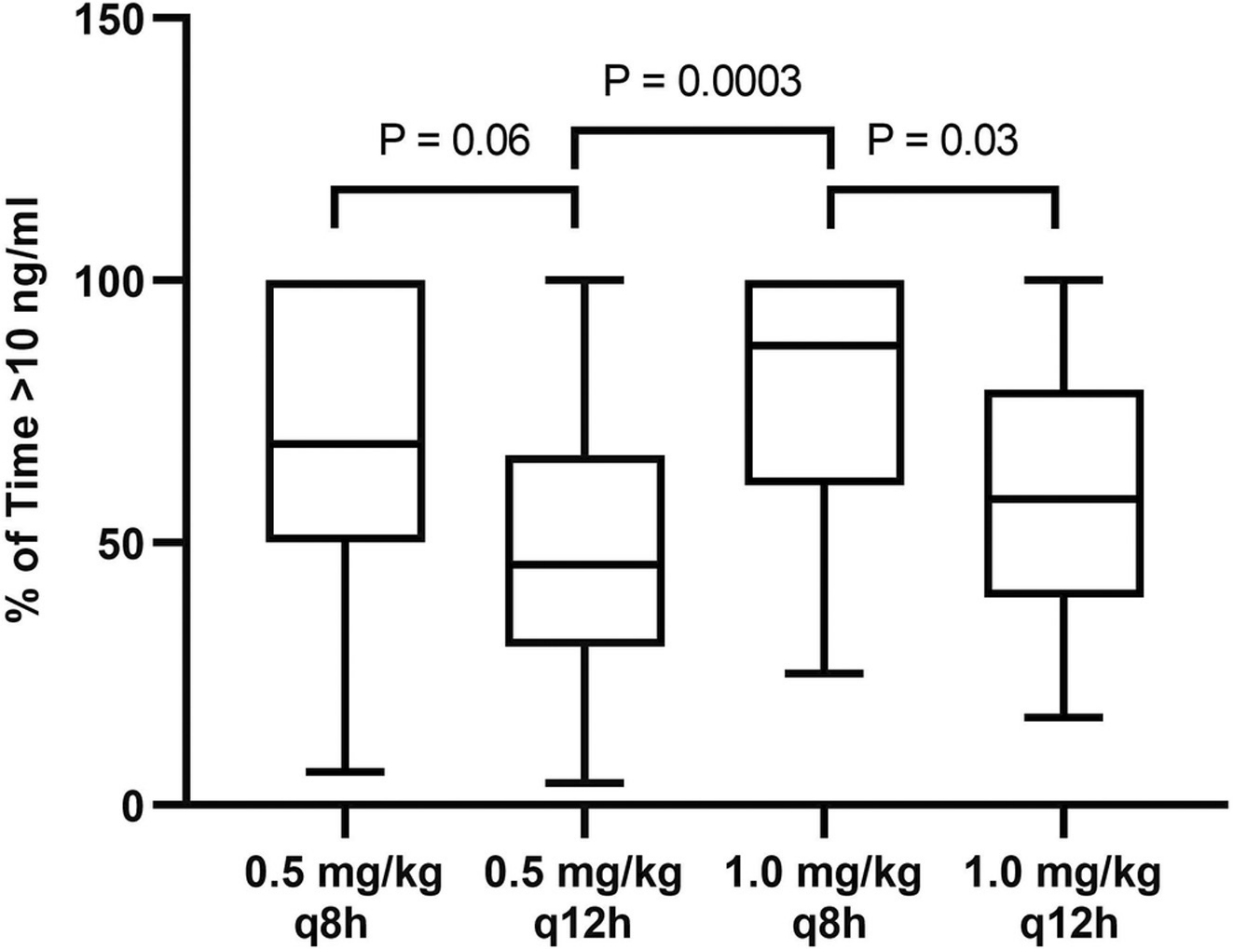
Dose interval modeling of different dosage regimens of ondansetron administered in hospitalized dogs exhibiting signs of nausea intravenously (*n* = 24). Values are presented as geometric mean ± geometric standard deviation.

Dose interval modeling is illustrated in Figure 1a–d. Based on this model, all four dosage regimens resulted in serum ondansetron concentrations within the therapeutic range; however, q8h dosing resulted in longer periods at higher serum concentrations. Differences between groups for duration of time above 10 ng/ml are presented in Figure 2. Dogs receiving 1 mg/kg q8h had statistically greater durations of time above 10 ng/ml as compared with dogs receiving 0.5 mg/kg q12h. Dogs receiving 1 mg/kg q8h had statistically greater durations of time above 10 ng/ml as compared with dogs receiving 1 mg/kg q12h. The linear (dose‐dependent) nature of IV ondansetron in this patient population supports the use of the PK data from both dose cohorts for dosing simulations at 0.5 and 1.0 mg/kg.

### 3.4 AVP concentrations

**FIGURE 3:**
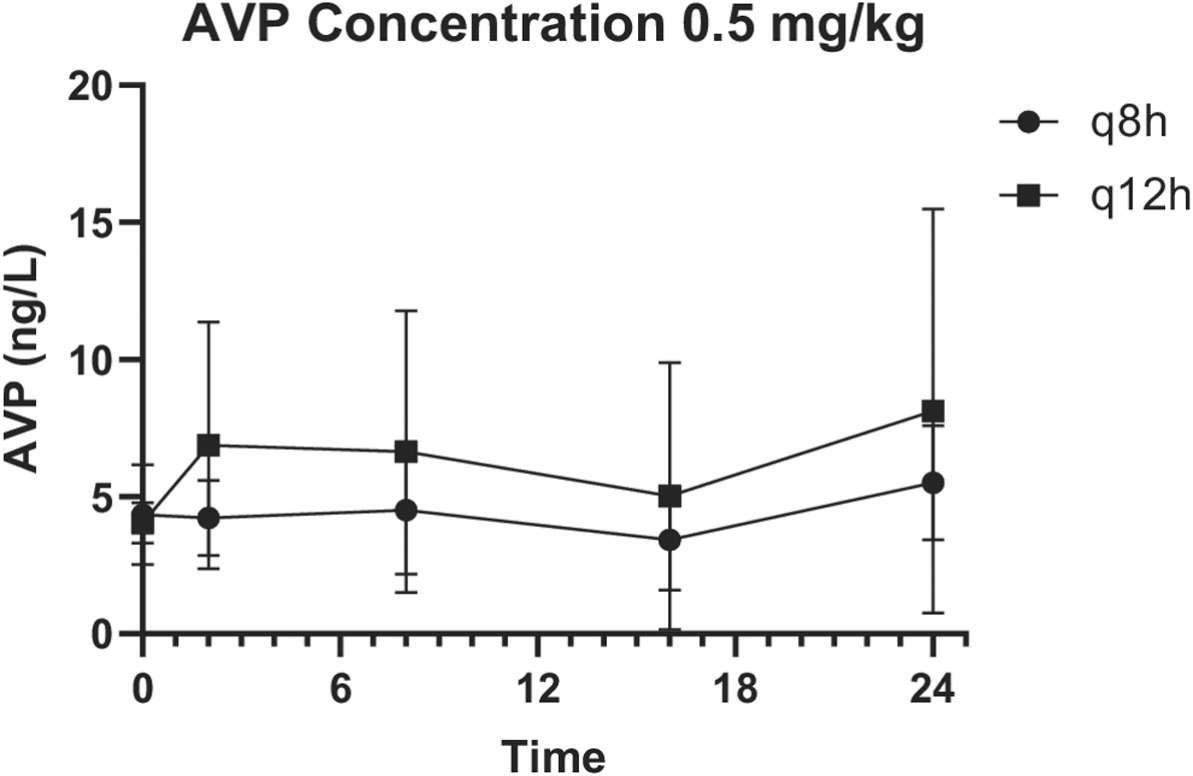
Mean serum arginine vasopressin (AVP) concentrations in hospitalized dogs exhibiting signs of nausea receiving 0.5 mg/kg ondansetron intravenously every 8 h or every 12 h. Values are presented as the mean ± standard deviation.

**FIGURE 4:**
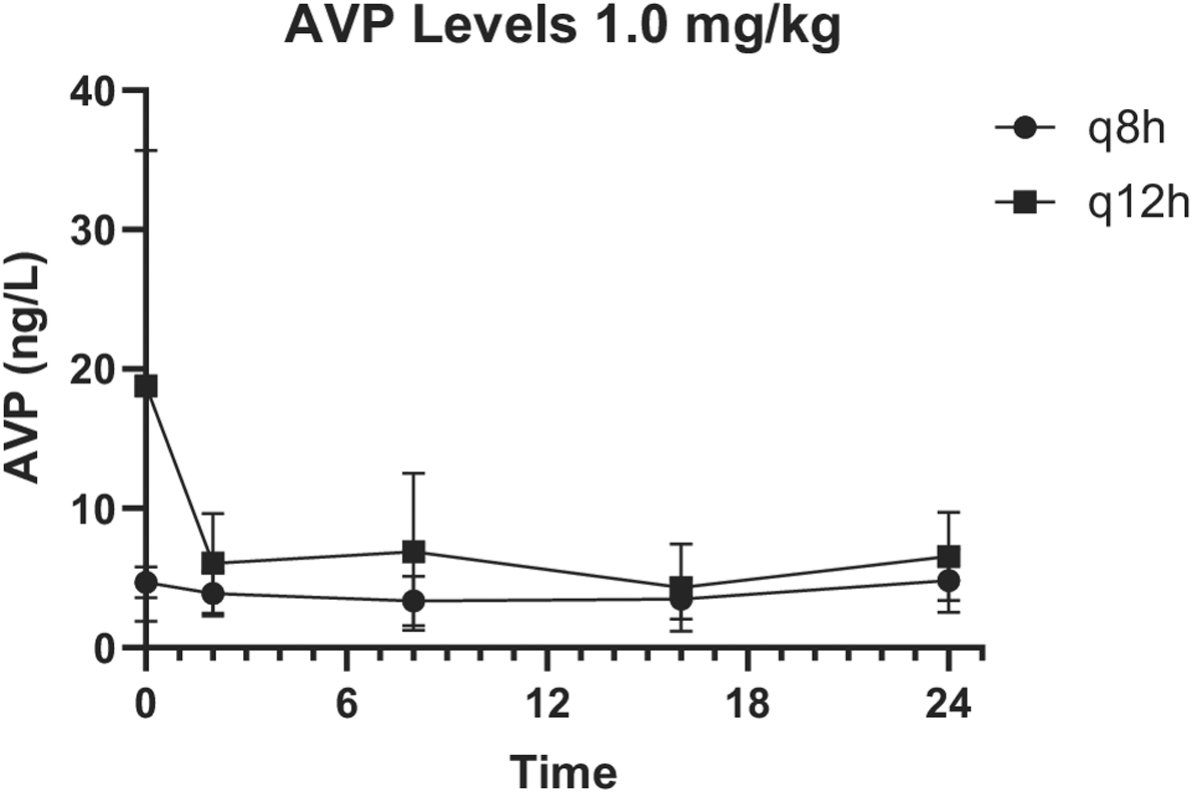
Mean serum arginine vasopressin (AVP) concentrations in hospitalized dogs exhibiting signs of nausea receiving 1 mg/kg ondansetron intravenously every 8 h or every 12 h. Values are presented as the mean ± standard deviation.

AVP concentrations were highly variable (Figures 3 and 4) and did not correlate with nausea scores (*p* = .54, Spearman *r* = .17).

AVP concentrations in the 0.5 mg/kg q12h group ranged from 2.3 to 21.2 ng/L, 0.5 mg/kg q8h group ranged from 1.79 to 8.8 ng/L, 1 mg/kg q12h group ranged from 2.8 to 25.3 ng/L, and 1 mg/kg q8h group ranged from 1.39 to 40.7 ng/L.

### 3.5 Nausea scores

**FIGURE 5:**
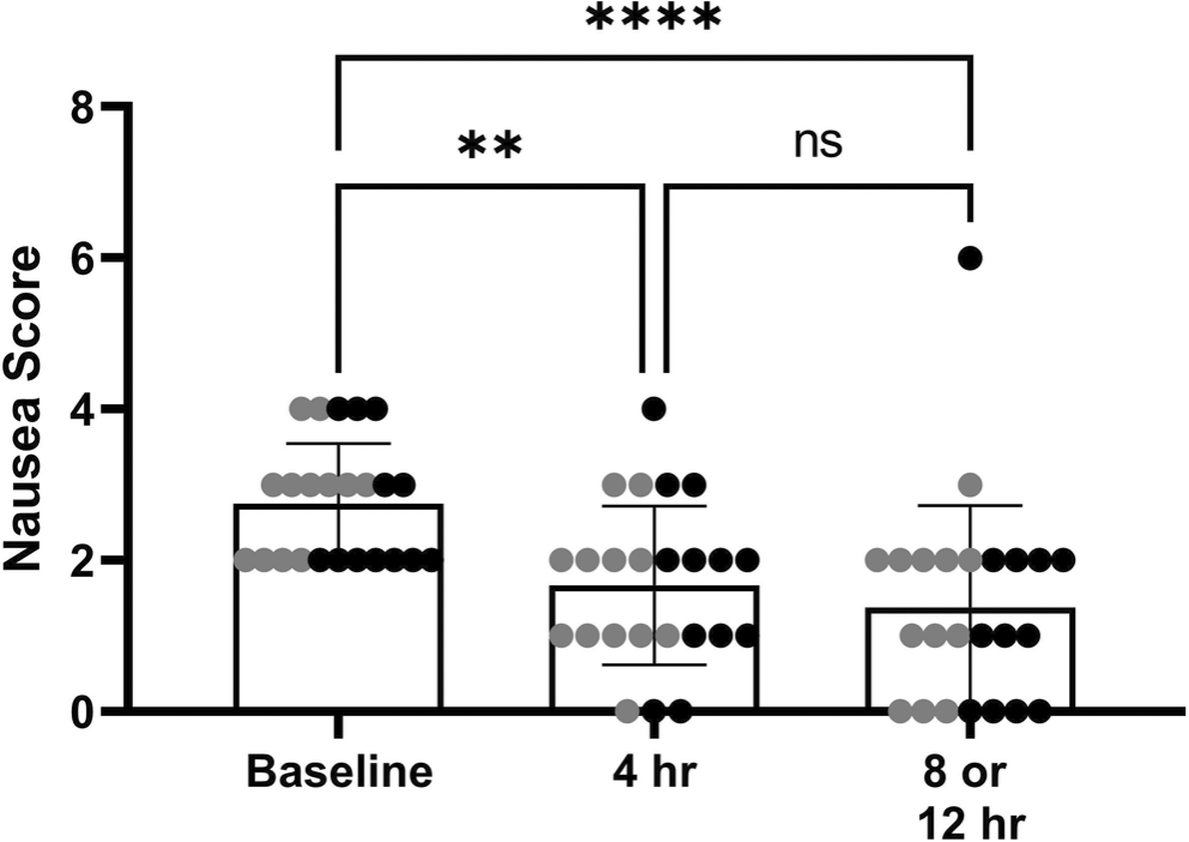
Nausea scores at 4 h and 8 or 12 h (based on dosing interval) compared with baseline nausea scores in all 24 hospitalized dogs receiving intravenous dosing of ondansetron. *denotes statistical significance. Values are presented as median (range). Gray circles represent dogs receiving ondansetron every 12 h, and black circles represent dogs receiving ondansetron every 8 h.

Mean baseline nausea scores were similar amongst groups. The baseline mean nausea scores in dogs receiving 0.5 mg/kg IV q8 and q12h were 2.5 (range 2–4) and 3.0 (range 2–4) respectively. Baseline mean nausea scores in dogs receiving 1 mg/kg IV q8 and q12h were 2.8 (range 2–4) and 2.6 (range 2–4), respectively. When all 24 dogs were considered together, mean nausea scores at 4 h (2; 0–4)(*p* = .002) and 8 or 12h (1; 0–6)(*p* < .0001) were significantly decreased from baseline (3; 2–4) (Figure 5).

### 3.6 Responders vs. non‐responders

There were eight non‐responders: one in the 0.5 mg/kg q8h group, two in the 0.5 mg/kg q12h group, two in the 1.0 mg/kg q8h group, and three in the 1.0 mg/kg q12h group. There was no statistical difference in the number of responders and non‐responders when comparing dogs receiving q8h or q12h dosing or when comparing patients receiving 0.5 mg/kg or 1 mg/kg. In our study, 83% of dogs (5/6) in the 0.5 mg/kg IV q8h group were classified as responders. The other dosing groups had a lower percentage of dogs that were classified as responders, specifically, 66.7% of dogs (4/6) in the 0.5 mg/kg q12h group and 1 mg/kg q8h group, and 50% of dogs (3/6) in the 1 mg/kg q12h group.

Using Fisher's exact test to compare dogs that received q12 h versus q8 h dosing, there was no statistical difference in whether a dog was classified as a responder or non‐responder. Dogs were just as likely to be classified as a responder regardless of the dose of ondansetron administered.

There was no statistical difference between the average cumulative ondansetron concentrations by 8 h in dogs that were defined as responders versus non‐responders regardless of dose given (0.5 or 1.0 mg/kg IV).

There was no statistical difference in clearance or *C*_max_ between responders and non‐responders.

## 4 DISCUSSION

In this study, ondansetron reached measurable serum concentrations in all dogs and displayed linear pharmacokinetics. This is particularly striking as dogs with a wide variety of disease processes, including renal and hepatic disease, were evaluated. Dogs were just as likely to be classified as a responder regardless of the dose of ondansetron administered, and there was no difference in pharmacokinetic parameters, such as clearance or *C*_max_, between responders and non‐responders. Based on dose interval modeling and extrapolating from a previous study (Kenward et al., 2017), serum concentrations of ondansetron were within the estimated therapeutic range for all dosage regimens in this study. Dogs that were receiving ondansetron q8h, regardless of the dose, had serum concentrations within the estimated therapeutic range for longer periods of time.

Nausea scores significantly decreased from baseline in all dogs treated with IV ondansetron. All four dose protocols appeared well tolerated and most dogs were considered responders. However, due to the small number of dogs in this study, an optimal dose cannot be recommended.

In this patient population, AVP concentrations did not correlate with severity of clinical signs as assessed by nausea scores. This is in contrast to a previous study (Kenward et al., 2017) where AVP increased after the administration of an emesis‐inducing chemotherapeutic drug. The prior study was performed in an experimental model versus our study, which included dogs with naturally occurring clinical signs of nausea. Our results may differ because cisplatin, a potent emetogen, was used in an experimental setting, whereas the dogs evaluated in this current study exhibited signs of nausea due to a variety of reasons such as gastrointestinal inflammation and severe systemic inflammation. Cisplatin targets the 5‐HT receptors allowing for both central and peripheral stimulation of nausea. It interacts with the enterochromaffin cells and the vagus nerves to stimulate nausea (Minami et al., 2003). In this case, one would expect that ondansetron would inhibit this pathway effectively and subsequently decrease AVP concentrations. The dogs in our study likely had a multimodal cause for nausea, not limited to just 5‐HT, but including gastric dysrhythmias, increases in substance P and dopamine (Singh et al., 2016). The patients in Kenward's study were hydrated with IV fluids, which may have resulted in lower baseline AVP concentrations, compared with our study, which included patients with varying hydration and volemic statuses. Additionally, the methodology by which AVP was assessed in this study (ELISA) also differs from the aforementioned study, which used radioimmunoassay and could also explain the contrasting results. Validation of this assay for serum was not performed in this study and should be considered a limitation. However, ELISA measurement of blood and saliva AVP has been recently validated in two studies assessing canine patients and proves to be a reliable way of measuring AVP (MacLean et al., 2017, 2018). The measurement of AVP using ELISA assays has become more common in several species as it does not require radioisotopes (Leng & Sabatier, 2016). Appropriate storage and handling of samples are imperative for accurate AVP measurement. All samples were handled in the same way in this study and were spun and stored at −80°C within 2 h of collection. Despite these efforts to mitigate any effects on samples, variability could have still affected our results. It should be also noted that stability of AVP during storage was not assessed in this study. No samples were stored for more than 9 months.

There were several other limitations to this study including the small number of dogs enrolled for evaluation of pharmacodynamics, and nausea scores were performed by multiple people versus a single observer. In addition, observers were not blinded to the dose randomization, which could have resulted in bias assigning nausea scores. In addition, there was variability in hydration status among the patients, which could have affected AVP measurement. Nausea scores decreased in all dogs regardless of the measured ondansetron concentrations, however, using nausea scores to assess response to therapy may be too simplified. The dogs in this study were receiving concurrent therapy for their primary disease, such as rehydration with IV fluids and antimicrobial therapy for suspected bacterial infections. The concurrent treatments may have contributed to the dogs having a decrease in nausea score. However, this is the type of heterogenous group of patients that are being evaluated every day in clinical practice and is more representative of the group of dogs that would be commonly prescribed ondansetron. Lastly, this study was not powered to detect a difference in response rate between doses.

In conclusion, intravenous ondansetron displays linear pharmacokinetics at the dosage protocols used in this study despite being evaluated in a mixed group of dogs with varying disease processes. All dogs had a decrease in nausea scores regardless of the dosage administered. AVP was variable and did not appear to be a reliable biomarker of nausea in this study. However, the true assessment of AVP as a suitable biomarker of nausea in the clinical setting could not be determined in this study based on the aforementioned limitations.

## AUTHOR CONTRIBUTIONS

CS, SShropshire, and KZ contributed to conception, experimental design, and analysis, drafted the manuscript, critically revised the manuscript, provided final approval, and agreed to be accountable for all aspects of work ensuring integrity and accuracy. JQ and DG contributed to experimental design and analysis, critically revised manuscript, and provided final approval. SSimpson contributed to experimental design, sample collection, critically revised the manuscript, and provided final approval.

## CONFLICT OF INTEREST

There are no conflicts of interest to disclose.

## ANIMAL WELFARE STATEMENT

The authors confirm that the ethical policies of the journal, as noted on the journal’s author guidelines page, have been adhered to. This study was approved by the Colorado State University Clinical Review Board and all owners signed a client consent form. The authors confirm that they have adhered to the US standards for the protection of animals used for scientific purposes.[Correction added on 30 August 2022, after first online publication: The Animal Welfare and Ethics Statement was included in this current version.]

## Data Availability

The data that support the findings of this study are available from the corresponding author upon reasonable request.

## References

[jvp13087-bib-0001] Baek, I.‐H. , Lee, B. Y. , Kang, J. , & Kwon, K. I. (2015). Pharmacokinetic modeling and Monte Carlo simulation of ondansetron following oral administration in dogs. Journal of Veterinary Pharmacology and Therapeutics, 38(2), 199–202. 25131428 10.1111/jvp.12147

[jvp13087-bib-0002] Cubeddu, L. X. , Lindley, C. M. , Wetsel, W. , Carl, P. L. , & Negro‐Vilar, A. (1990). Role of angiotensin II and vasopressin in cisplatin‐induced emesis. Life Sciences, 46(10), 699–705. 2314191 10.1016/0024-3205(90)90075-3

[jvp13087-bib-0003] Foth, S. , Meller, S. , Kenward, H. , Elliott, J. , Pelligand, L. , & Volk, H. A. (2021). The use of ondansetron for the treatment of nausea in dogs with vestibular syndrome. BMC Veterinary Research, 17(1), 1–9. 34154584 10.1186/s12917-021-02931-9 PMC8218477

[jvp13087-bib-0004] Kenward, H. , Elliott, J. , Lee, T. , & Pelligand, L. (2017). Anti‐nausea effects and pharmacokinetics of ondansetron, maropitant and metoclopramide in a low‐dose cisplatin model of nausea and vomiting in the dog: A blinded crossover study. BMC Veterinary Research, 13(1), 1–12. 28814338 10.1186/s12917-017-1156-7 PMC5559813

[jvp13087-bib-0005] Kenward, H. , Pelligand, L. , Savary‐Bataille, K. , & Elliott, J. (2015). Nausea: Current knowledge of mechanisms, measurement and clinical impact. The Veterinary Journal, 203(1), 36–43. 25453240 10.1016/j.tvjl.2014.10.007

[jvp13087-bib-0006] Kretzing, S. , Abraham, G. , Seiwert, B. , Ungemach, F. R. , Krügel, U. , Teichert, J. , & Regenthal, R. (2011). In vivo assessment of antiemetic drugs and mechanism of lycorine‐induced nausea and emesis. Archives of Toxicology, 85(12), 1565–1573. 21626407 10.1007/s00204-011-0719-9

[jvp13087-bib-0007] Leng, G. , & Sabatier, N. (2016). Measuring oxytocin and vasopressin: Bioassays, immunoassays and random numbers. Journal of Neuroendocrinology, 28, 10. 10.1111/jne.12413 PMC5096068 27467712

[jvp13087-bib-0008] MacLean, E. L. , Gesquiere, L. R. , Gee, N. , Levy, K. , Martin, W. L. , & Carter, C. S. (2018). Validation of salivary oxytocin and vasopressin as biomarkers in domestic dogs. Journal of Neuroscience Methods, 293, 67–76. 28865986 10.1016/j.jneumeth.2017.08.033

[jvp13087-bib-0009] MacLean, E. L. , Gesquiere, L. R. , Gruen, M. E. , Sherman, B. L. , Martin, W. L. , & Carter, C. S. (2017). Endogenous oxytocin, vasopressin, and aggression in domestic dogs. Frontiers in Psychology, 8, 1613. 29021768 10.3389/fpsyg.2017.01613 PMC5624304

[jvp13087-bib-0010] Minami, M. , Endo, T. , Hirafuji, M. , Hamaue, N. , Liu, Y. , Hiroshige, T. , Nemoto, M. , Saito, H. , & Yoshioka, M. (2003). Pharmacological aspects of anticancer drug‐induced emesis with emphasis on serotonin release and vagal nerve activity. Pharmacology & Therapeutics, 99(2), 149–165. 12888110 10.1016/s0163-7258(03)00057-3

[jvp13087-bib-0011] Pirrone, F. , Pierantoni, L. , Bossetti, A. , Uccheddu, S. , & Albertini, M. (2019). Salivary vasopressin as a potential non–invasive biomarker of anxiety in dogs diagnosed with separation–related problems. Animals, 9(12), 1033. 31779267 10.3390/ani9121033 PMC6941168

[jvp13087-bib-0012] Plumb, D. C. (2019). *Ondansetron. Plumb's Veterinary Drugs*. https://app.plumbs.com/drug‐monograph/vTu5mODx1yPROD

[jvp13087-bib-0013] Quimby, J. M. , Lake, R. C. , Hansen, R. J. , Lunghofer, P. J. , & Gustafson, D. L. (2014). Oral, subcutaneous, and intravenous pharmacokinetics of ondansetron in healthy cats. Journal of Veterinary Pharmacology and Therapeutics, 37(4), 348–353. 10.1111/jvp.12094 24330064 PMC4059788

[jvp13087-bib-0014] Sedlacek, H. S. , Ramsey, D. S. , Boucher, J. F. , Eagleson, J. S. , Conder, G. A. , & Clemence, R. G. (2008). Comparative efficacy of maropitant and selected drugs in preventing emesis induced by centrally or peripherally acting emetogens in dogs. Journal of Veterinary Pharmacology and Therapeutics, 31(6), 533–537. 19000276 10.1111/j.1365-2885.2008.00991.x

[jvp13087-bib-0015] Seynaeve, C. , Schuller, J. , Buser, K. , Porteder, H. , Van Belle, S. , Sevelda, P. , Christmann, D. , Schmidt, M. , Kitchener, H. , & Paes, D. (1992). Comparison of the anti‐emetic efficacy of different doses of ondansetron, given as either a continuous infusion or a single intravenous dose, in acute cisplatin‐induced emesis. A multicentre, double‐blind, randomized, parallel group study. British Journal of Cancer, 66(1), 192–197. 1386245 10.1038/bjc.1992.241 PMC1977922

[jvp13087-bib-0016] Singh, P. , Yoon, S. S. , & Kuo, B. (2016). Nausea: A review of pathophysiology and therapeutics. Therapeutic Advances in Gastroenterology, 9(1), 98–112. 10.1177/1756283X15618131 26770271 PMC4699282

[jvp13087-bib-0017] Sullivan, L. A. , Lenberg, J. P. , Boscan, P. , Hackett, T. B. , & Twedt, D. C. (2018). Assessing the efficacy of maropitant versus ondansetron in the treatment of dogs with parvoviral enteritis. Journal of the American Animal Hospital Association, 54(6), 338–343. 30272481 10.5326/JAAHA-MS-6650

